# TAF6δ orchestrates an apoptotic transcriptome profile and interacts functionally with p53

**DOI:** 10.1186/1471-2199-11-10

**Published:** 2010-01-22

**Authors:** Emmanuelle Wilhelm, Mara Kornete, Brice Targat, Jimmy Vigneault-Edwards, Mattia Frontini, Laszlo Tora, Arndt Benecke, Brendan Bell

**Affiliations:** 1 RNA Group. Département de microbiologie et d'infectiologie, Faculté de médecine et sciences de la santé, Université de Sherbrooke, 3001 12e ave Nord, Sherbrooke, Québec J1H 5N4, Canada; 2 Institut des Hautes Études Scientifiques & Institut de Recherche Interdisciplinaire - CNRS/USTL; 35 route de Chartres; 91440 Bures sur Yvette, France; 3 Clinical Science Center, Hammersmith Hospital Campus, Du Cane Road, W12 0NN, London, UK; 4 Department of Functional Genomics Institut de Génétique et de Biologie Moléculaire et Cellulaire (IGBMC), UMR 7104 CNRS, UdS, INSERM U964, BP 10142, F-67404 ILLKIRCH Cedex, CU de Strasbourg, France

## Abstract

**Background:**

TFIID is a multiprotein complex that plays a pivotal role in the regulation of RNA polymerase II (Pol II) transcription owing to its core promoter recognition and co-activator functions. TAF6 is a core TFIID subunit whose splice variants include the major TAF6α isoform that is ubiquitously expressed, and the inducible TAF6δ. In contrast to TAF6α, TAF6δ is a pro-apoptotic isoform with a 10 amino acid deletion in its histone fold domain that abolishes its interaction with TAF9. TAF6δ expression can dictate life versus death decisions of human cells.

**Results:**

Here we define the impact of endogenous TAF6δ expression on the global transcriptome landscape. TAF6δ was found to orchestrate a transcription profile that included statistically significant enrichment of genes of apoptotic function. Interestingly, gene expression patterns controlled by TAF6δ share similarities with, but are not equivalent to, those reported to change following TAF9 and/or TAF9b depletion. Finally, because TAF6δ regulates certain p53 target genes, we tested and demonstrated a physical and functional interaction between TAF6δ and p53.

**Conclusion:**

Together our data define a TAF6δ-driven apoptotic gene expression program and show crosstalk between the p53 and TAF6δ pathways.

## Background

Apoptosis is an active program of cell death that is required for normal development and tissue homeostasis in metazoans [1]. The deregulation of apoptotic pathways underlies many human diseases [2]. Consequently, apoptotic pathways represent potential targets for therapeutic control of cell death for diseases including neurodegenerative disorders, autoimmune diseases and cancer [3]. Our previous studies have uncovered the existence of an apoptotic pathway termed the TAF6δ pathway that controls cell death [4,5].

TAF6δ is an inducible splice variant of the TFIID subunit TAF6 (previously termed hTAF_II_70 or hTAF_II_80). TFIID is a multiprotein complex containing the TATA-binding protein (TBP) and up to 14 evolutionarily conserved TBP-associated factors (TAFs) [6,7]. TFIID is the primary core promoter recognition complex for RNA polymerase II (pol II) and thus plays a key role in the regulation of transcription of protein-coding genes [8]. The major TAF6α isoform is ubiquitously expressed [9] whereas strong expression of the TAF6δ isoform has only been detected in apoptotic conditions (e.g. HL-60 cells undergoing retinoic acid dependent death) [4]. The use of modified antisense RNA oligonucleotides, also termed splice-switching oligonucleotides (SSO), to experimentally direct the expression of endogenous TAF6δ in living cells has recently demonstrated the pro-apoptotic activity of TAF6δ [5].

The major TAF6α isoform contributes to the stability of core TFIID complexes in part by dimerizing with TAF9 via its histone fold domain [9-13]. Structurally, TAF6δ differs from TAF6α only in that it lacks 10 amino acids within its histone fold domain. These amino acids, however, are critical for the interaction of TAF6α with TAF9 [14], and as a consequence, TAF6δ cannot interact with TAF9 [4]. As is the case for TAF9, the highly homologous protein TAF9b cannot interact with the pro-apoptotic TAF6δ isoform [15]. TAF6δ does retain the capacity to interact directly with other TFIID subunits including TAF1, TAF5, TBP and TAF12. Consequently, within cells TAF6δ is incorporated into a TFIID-like complex that lacks TAF9 and TAF9b, termed TFIIDπ [4]. Depletion of TAF9 or the highly homologous protein TAF9b in HeLa cells has been shown to alter global gene expression patterns [16]. Presently it is not known whether the transcriptional effects of TAF6δ are related to those resulting from the depletion of TAF9 and/or TAF9b. Our previous work revealed that TAF6δ can alter gene expression [5], but a physiologically informative definition of the transcriptome impact of TAF6δ is currently lacking.

Data documenting a direct interaction between the major TAF6α isoform with p53 has been shown *in vitro *using recombinant proteins [17], *in vitro *using endogenous human TFIID [18], and in cultured cells using reporter assays [19]. Furthermore, the interaction of TAF6α with p53 has been shown to be essential for the activation of transcription by p53 *in vitro *[17] as well as *in vivo *in mice bearing point mutations within p53 that block its interaction with TAF6α [20]. Currently it is not known whether the inducible pro-apoptotic TAF6δ isoform can interact with p53. Importantly, TAF6δ induces apoptosis in cell lines that lack p53 expression [5]. Moreover, the induction of TAF6δ produced similar levels of apoptosis in the HCT-116 p53 -/- colon carcinoma cell line as in its p53 positive counterpart [5]. Thus, TAF6δ can induce programmed cell death independently of p53, however the functional relationship between the TAF6δ and p53 pathways requires further clarification.

The TAF6δ pathway represents a tractable experimental paradigm to elucidate the mechanisms by which human cells respond to their environment through subunit changes in the general transcription machinery [21]. Moreover, there is mounting evidence that the TAF6δ pathway may be altered in certain cancers. Aberrant TAF6 expression has been documented in human cancers including lung cancer [22,23] and breast cancer [24,25]. The molecular basis for the induction of apoptosis by TAF6δ is currently unknown. In order to shed further light on the impact of TAF6δ on the human transcriptome, here we performed a transcriptome-wide analysis of the impact of endogenous TAF6δ expression in HeLa cervical carcinoma cells. Our data provide the first physiologically coherent transcriptome signature for TAF6δ, establish the relationship of the TAF6δ signature with those of TAF9/TAF9b, and identify a functional and physical interaction of TAF6δ with p53.

## Results

### The TAF6δ orchestrates a pro-apoptotic gene expression program

To establish the impact of TAF6δ on global gene expression patterns, the expression of the endogenous TAF6δ splice variant was experimentally induced using splice-switching oligonucleotides (SSO) as previously documented [5]. The HeLa cell line was chosen as a model system for transcriptome studies for three principle reasons. Firstly, these cells are readily transfectable and produce a robust apoptotic response to TAF6δ-inducing SSO [5]. Secondly, the TAF6δ cDNA was cloned from a HeLa cell library [4] and therefore these cells provide a natural cellular context. Thirdly, HeLa cells express no detectable TAF6δ protein under standard culture conditions [5], thus these cells provide a stringently inducible model for SSO studies. To define the impact of TAF6δ expression on transcriptome dynamics we took advantage of an experimental approach that combines SSO treatment with high sensitivity microarray analysis [26]. We note that to achieve the statistically significant overrepresentation of gene ontology pathways reported here it was necessary to employ optimized SSO sequences designed to more efficiently induce TAF6δ expression than those employed in a previous study [5]. The improved SSO were transfected into HeLa cells and the induction of endogenous TAF6δ mRNA and protein was confirmed, as shown in Additional File 1. Total RNA was isolated 18 hours post-transfection and subjected to microarray analysis as previously detailed [26]. Biological triplicates were performed with SSO T6-1 and, as a control to normalize for any non-specific SSO effects, a scrambled oligonucleotide (SSO ctrol). The statistical analysis and filtering of the raw microarray data was carried out as previously described [26] to identify significantly (P < 0.05) regulated mRNAs.

**Figure 1:**
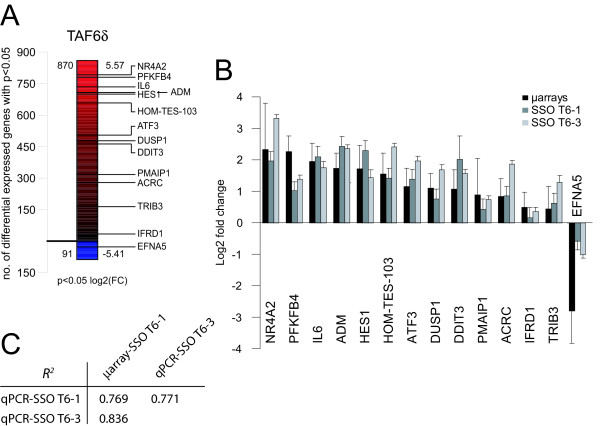
**Transcriptome analysis following SSO induction of TAF6δ**. (A) Expression levels of mRNAs from T6-1 SSO-treated HeLa cells were individually compared to those from control oligonucleotide-treated HeLa samples by genome-wide microarray analysis. The absolute number of probes detecting statistically significant (P < 0.05) up- or down-regulation following TAF6δ induction is shown to the left of each bar; the positive or negative logarithmic (base two) fold-change is shown to the right. The red gradient indicates positive, and the blue gradient negative fold changes in expression. The relative position of the TAF6δ-regulated genes that were further validated by qPCR (panel B) is indicated on the right. (B) Independent verification of gene expression changes by quantitative real-time RT-PCR. HeLa cells were transfected with SSO T6-1 (dark grey bars) or SSO T6-3 (light grey bars) to induce endogenous TAF6δ. 18 hours post-transfection total RNA was analyzed by quantitative real-time PCR and compared with microarray measurements from HeLa cells transfected with SSO T6-1 (black bars). Error bars indicate standard deviation of three independent transfections. (C) The Pearson correlation coefficients associated with panel B.

The induction of endogenous TAF6δ resulted in significant changes in the levels of 961 probes corresponding to 955 independent genes of 27,868 (Figure 1A). Remarkably, 90.5% of the mRNAs significantly changed by TAF6δ are upregulated and only 9.5% are downregulated (Figure 1A). These data are consistent with previous results obtained with a less efficient SSO in the HCT-116 cell line [5] and further demonstrate that TAF6δ acts primarily as a positive regulator of gene expression. The data also rule out the possibility that TAF6δ-induced cell death is a result of a global reduction in mRNA transcription. To validate the TAF6δ transcriptome signature we selected 14 genes (and the internal control beta-2-microglobulin gene) for quantitative RT-PCR confirmation. Gene expression changes measured by real-time RT-PCR for the 14 genes showed a strong correlation with changes measured by microarray analysis (Pearson correlation coefficient *R*^2 ^= 0.769, Figure 1B &1C). To further confirm the specificity of the TAF6δ transcriptome signature we employed a distinct TAF6 targeting antisense oligonucleotide (SSO T6-3), whose binding to the TAF6 pre-mRNA is shifted five nucleotides downstream with respect to SSO T6-1. SSO T6-3 is slightly more efficient in inducing endogenous TAF6δ than SSO T6-1 (Additional File 1). Real-time RT-PCR shows that like SSO T6-1, SSO T6-3 also altered the expression of the same 14 TAF6δ target genes (Pearson correlation coefficient with microarray measurements *R*^2 ^= 0.836, Figure 1B &1C). The above data confirm that the microarray results provide an accurate and reproducible measure of the TAF6δ-controlled transcriptome landscape.

To examine the specificity of the TAF6δ-induced transcriptome signature, we compared the microarray data with those obtained when the pro-apoptotic isoform of a distinct gene, Bcl-x, was induced by SSO under identical conditions [5]. As shown in Additional File 2, the transcriptome signatures resulting from the induction of TAF6δ and Bcl-xS are highly distinct. The vast majority of TAF6δ-regulated transcripts (90.5%) were induced while Bcl-xS expression results in a majority of transcripts being repressed (58%). Only a minor fraction (3.4%) of the 870 genes upregulated by TAF6δ was also upregulated by Bcl-xS. Of the small number of transcripts repressed (46) by TAF6δ, 45 are also repressed by Bcl-xS, possibly reflecting a minor subset of genes that are repressed by both of these pro-apoptotic pathways. The portion of Bcl-xS repressed genes also repressed by TAF6δ was minor (15.8%). The fact that genes induced by TAF6δ share little overlap with those induced by Bcl-xS underscores the highly specific impact of the TAF6δ-inducing SSO on the transcriptome.

**Figure 2:**
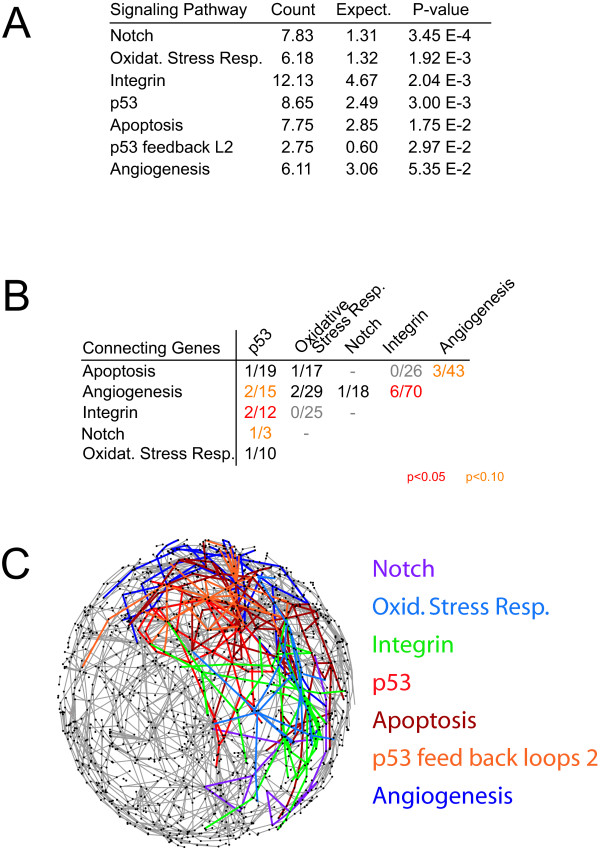
**Pathway analysis of the TAF6δ transcriptome signature**. (A) Specific cellular pathways are statistically significantly (P < 0.05) overrepresented in the TAF6δ-regulated transcriptome. "Count" indicates the number of probes within the dataset that corresponds to a given gene ontology pathway, "Expect." is the number of probes one would expect to find for a given pathway in a random dataset of identical size. (B) Interconnectivity analysis reveals statistically significant overrepresentation of shared genes between TAF6δ-affected cellular pathways. Red and orange indicate highly significant (P < 0.05) and significant (P < 0.10) overrepresentation of shared regulated genes, light grey indicates present links of low statistical significance between the pathways from B. (C) Interconnectivity between overrepresented pathways (highlighted in color and listed at right) is shown in a network representation generated using Pajek software [27].

To shed light on the mechanisms underlying the pro-apoptotic capacity of TAF6δ, we performed gene ontology analysis to identify pathways that are statistically overrepresented within the microarray data (see details in Materials and Methods). Of 131 cellular pathways surveyed, those that are statistically (P < 0.05) overrepresented in the TAF6δ data set are the Notch, oxidative stress response, integrin, p53, apoptosis and p53 pathway feedback loops 2 pathways (Figure 2A). Genes in the angiogenesis pathway were also overrepresented (P = 0.0535, Figure 2A) in the TAF6δ-induced gene pool. The TAF6δ-regulated genes found in the overrepresented pathways are shown in Additional File 3. We next postulated that if the pathways activated by TAF6δ represent a physiologically coherent response, then the functional connections between these pathways would not be random. We therefore performed a statistical analysis to identify pathways that share two or more genes activated by TAF6δ at frequencies higher than those expected in a random sample. Statistically significant (P < 0.05) overrepresentation of shared genes between the integrin and angiogenesis pathways, as well as between the p53 and integrin pathways was observed (Figure 2B). To provide a visual framework that depicts the interconnections between the overrepresented pathways they were mapped onto cellular signaling networks using the Pajek algorithm [27]. This global view reveals that the activated pathways are not randomly distributed and moreover that there is a network of interconnections between TAF6δ-regulated pathways, as seen by black points at which distinct colored lines intersect (Figure 2C). Taken together, the results above define a specific TAF6δ-driven transcriptome landscape that includes the induction of genes in the Notch, oxidative stress response, integrin, p53, apoptosis, p53 pathway feedback loops 2 and angiogenesis pathways.

**Figure 3:**
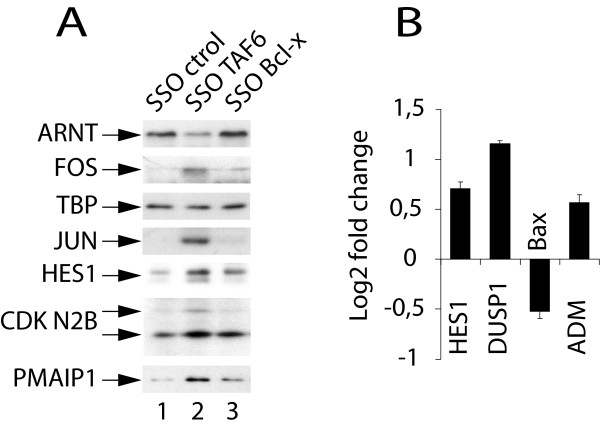
**TAF6δ controls protein expression and promoter-dependent transcription**. (A) HeLa cells were treated with control (ctrol), TAF6δ-inducing (SSO T6-3), or Bcl-xS-inducing (SSO Bcl-x) splice-switching oligonucleotides. Total protein extracts were prepared 18 hours post-transfection and fractionated by SDS-PAGE followed by immunoblot analysis of endogenous protein levels with antibodies indicated at the left. (B) Promoter-dependent regulation of transcription by endogenous TAF6δ. HeLa cells were transfected with 100 to 200 ng of HES1, DUSP1, BAX or ADM promoter-luciferase constructs. 24 hours post-transfection the cells were treated with 100 nM TAF6δ-inducing oligonucleotide for another 24 hours before measurement of luciferase activity (y-axis). Results are expressed as log_2 _fold change of TAF6δ SSO versus control SSO treated cells. Error bars indicate standard deviation of three independent transfections.

To determine whether or not the changes in mRNA expression in response to TAF6δ expression result also in changes in protein levels we selected representative proteins from the TAF6δ transcriptome signature for verification by immunoblotting experiments. The microarray data showed expression of ARNT mRNA was repressed by TAF6δ, and immunoblotting showed the corresponding ARNT protein levels also decreased in response to TAF6δ-inducing SSO (Figure 3A). The levels of the transcription factor TBP were tested as a control for specificity and remained relatively constant in response to TAF6δ (Figure 3A). The levels of FOS, JUN, HES1, CDKN2B (p15INK4B), and PMAIP1 (NOXA) were assayed and TAF6δ expression resulted in increased protein levels that paralleled increased mRNA levels detected in the microarray experiments in each case (Figure 3A). Importantly, Bcl-x SSO treatment did not result in comparable changes of protein levels, showing the specificity of their response to TAF6δ expression (Figure 3A). These data demonstrate that for the proteins tested the impact of TAF6δ on gene expression programs occurs at both the mRNA and protein levels, including the induction of the known pro-apoptotic protein PMAIP1 (NOXA) [28].

Changes in mRNA levels detected by microarray analysis can in principle result from a number of effects including alterations in mRNA stability. To obtain evidence that TAF6δ can regulate gene expression in a promoter-dependent and promoter-specific fashion, we tested the ability of endogenous TAF6δ to increase target gene expression in luciferase reporter gene assays. We selected four promoters for analysis. The HES1, DUSP1 and ADM promoters were selected since the endogenous *HES1*, *DUSP1 *and *ADM *mRNA levels are induced in response to TAF6δ expression (Figure 1A &1B). In the case of HES1, the levels of endogenous HES1 protein were also shown to be induced in response to TAF6δ expression (Figure 3A). The 3 selected genes act in several of the pathways activated by TAF6δ including the Notch (HES1) [29], angiogenesis (ADM) [30], oxidative stress and p53 pathways (DUSP1) [31-33]. The Bax promoter was also included because it is a p53-responsive and pro-apoptotic gene [34], yet is not induced by TAF6δ. The induction of TAF6δ in HeLa cells resulted in increased HES1, DUSP1, and ADM promoter-driven gene expression (Figure 3B). In contrast, the Bax promoter was not induced and even measurably repressed (Figure 3B). These results demonstrate that endogenous TAF6δ can act directly or indirectly to stimulate transcription in a promoter-dependent manner.

### The TAF6δ transcriptome signature is not equivalent to those resulting from depletion of TAF9 and/or TAF9b

**Figure 4:**
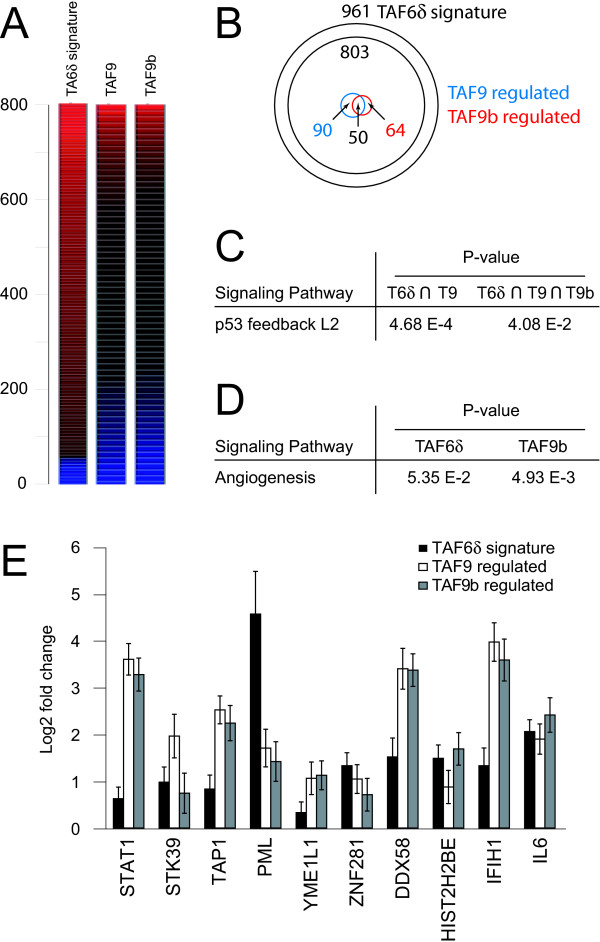
**Comparison of the transcriptome effects of TAF6δ with TAF9 and TAF9b**. (A) A heat map representation of changes in expression of 800 mRNAs that could be mapped from the published data resulting from depletion of TAF9 or TAF9b [16] to the TAF6δ-dependent transcriptome signature shows the global comparison of their respective transcriptome profiles. The red gradient indicates positive, and the blue gradient negative fold changes in expression. (B) A Venn diagram representation of the transcriptome comparison shows the relationship between genes significantly regulated by TAF6δ, TAF9 and TAF9b. 803 probes were mapped onto the 961 probe TAF6δ signature. (C) Overrepresentation of the "p53 feedback loop 2" gene ontology pathway is common to the TAF6δ, TAF9 and TAF9b signatures. Pathway analysis was performed as described in Materials and Methods for genes significantly regulated by both TAF6δ and TAF9 (left), by all of TAF6δ, TAF9 and TAF9b (right). P-values for overrepresentation are shown in the table. (D) Overrepresentation of the angiogenesis gene ontology pathway is common to the TAF6δ, TAF9 and TAF9b transcription signatures. Pathway analysis as in (C). (E) Microarray recorded gene expression changes for examples of genes similarly regulated by TAF6δ, TAF9 and TAF9b are shown. Error bars indicate standard deviations.

The only currently known functional distinction between pro-apoptotic TAF6δ and TAF6α is that TAF6δ cannot interact with TAF9 or TAF9b. We therefore analyzed the extent to which the transcription footprints resulting from TAF6δ induction resembles those reported following the depletion of TAF9 and/or TAF9b by treatment with siRNAs in HeLa cells [16]. After mapping of the previous microarray data to enable comparison with our current microarray platform (see Materials and Methods), the datasets were co-filtered to compare transcriptome changes. Of the 961 TAF6δ-dependent mRNAs selected for comparative analysis, 803 mRNAs could be mapped between the datasets (Figure 4B). A global view of the magnitude of the changes for these 803 genes showed that changes in response to TAF6δ induction were more pronounced than those resulting from TAF9/TAF9b depletion as depicted by heat maps (Figure 4A). 204 mRNAs that are statistically significantly regulated by TAF9 and/or TAF9b were found within the TAF6δ-regulated transcripts (Figure 4B). Of the 204 genes, 50 showed regulation by both TAF9 and TAF9b. 90 showed regulation by TAF9 alone and 64 showed regulation by TAF9b alone (Figure 4B). To probe for communalities between TAF6δ, TAF9 and TAF9b-controlled transcriptomes, pathway analysis was performed on the genes regulated by TAF6δ and TAF9 as well as genes regulated by TAF6δ, TAF9, and TAF9b. The only pathway that was statistically significantly overrepresented in these subsets was the "p53 feedback loop 2" ontology (Figure 4C). Pathway analysis was also performed on the TAF9b-dependent gene set alone. Interestingly, the only pathway that was overrepresented was angiogenesis, a pathway also overrepresented in the TAF6δ transcriptome signature (Figure 4D). Finally, we compared the effects of TAF6δ, TAF9, and TAF9b on single genes from the subset of 50 genes that respond to changes in the expression all of TAF6δ, TAF9, and TAF9b. A majority of genes (~80%) had comparable changes in response to TAF6δ induction and TAF9 or TAF9b depletion of which 10 examples are shown in Figure 4D. In contrast, approximately 20% of the TAF6δ- TAF9- and TAF9b-dependent genes were positively regulated by TAF6δ induction whereas TAF9 or TAF9b depletion resulted in their suppression (Additional File 4). Taken together the analysis shows that TAF6δ induction has both overlapping and unique impacts on the transcriptome, when compared to TAF9 and/or TAF9b depletion.

### TAF6δ interacts physically and functionally with p53

**Figure 5:**
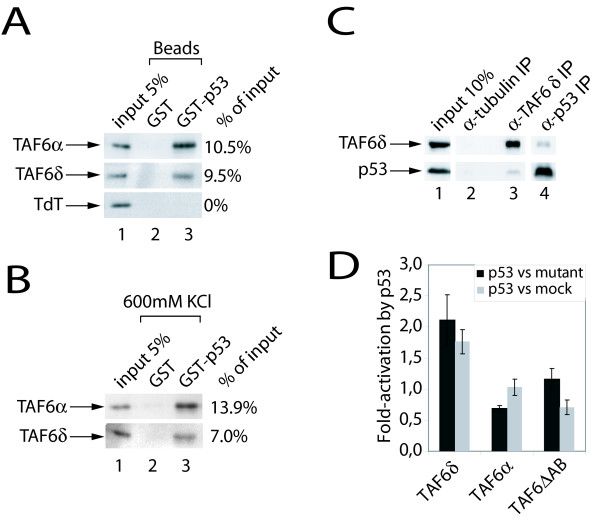
**TAF6δ interacts physically and functionally with p53**. (A) TAF6δ interacts with p53 *in vitro*. Recombinant purified TAF6α, TAF6δ, or TdT were incubated with immobilized GST-p53. Complexes were washed and retained proteins were analyzed by Western blot. The percentage of retention as quantified by phosphoimager analysis is given at the right. (B) Complexes were washed as in (A) except that the washing buffer contained 600 mM KCl. (C) TAF6δ interacts with p53 *in vivo*. HCT116 p53 -/- cells were transfected with plasmids expressing TAF6δ and p53. 28 hours post-transfection protein interactions were assayed by co-immunoprecipitation followed by Western blotting with the appropriate antibodies (Materials and Methods). (D) TAF6δ enhances p53-mediated activation of the DUSP1 promoter. HeLa cells were co-transfected with a plasmid expressing the firefly luciferase gene under control of DUSP1 promoter, a plasmid expressing p53 or its mutant R175H or no p53 and various constructs of TAF6. 28 h after transfection, cells were lysed and luciferase activity was measured. Shown are the ratios of relative light unit (RLU) given by cells transfected with p53 relative to cells transfected with p53R175H (black bars) or no p53 (grey bars).

TAF6α is known to interact with the p53 tumor suppressor protein (see Introduction), but whether the pro-apoptotic TAF6δ isoform could retain the capacity to interact with p53 is unknown. Our previous work demonstrated that TAF6δ induces apoptosis independently of p53 [5]. Interestingly, the current transcriptome data show that TAF6δ induces the expression of several p53 target genes (Figure 2A and Additional File 3). We therefore investigated the capacity of TAF6δ to interact with p53. Recombinant Histidine tagged TAF6δ was produced in bacteria and purified by nickel affinity chromatography. GST tagged p53 was produced and immobilized on glutathione-sepharose beads. Purified recombinant TAF6δ and TAF6α were assayed for their capacities to interact with immobilized GST-p53. As previously reported [17], TAF6α bound efficiently to GST-p53 (Figure 5A, lane 3, upper row). Terminal deoxynucleotidyl transferase (TdT) served as a control for specificity and did not bind to GST-p53 (Figure 5A, lane 3, lower row). Purified His-TAF6δ was efficiently retained by GST-p53 (Figure 5A, lane 3, middle row), but not by GST alone (Figure 5A, lane 2). Resistance to high ionic strength buffers can provide a measure of the strength of protein-protein interactions. We therefore challenged the p53-TAF6 interactions with 600 mM KCl washes and found that the interactions were stable in high salt conditions (Figure 5B). These data show a direct and selective interaction between TAF6δ and p53 *in vitro*.

To determine whether the TAF6δ-p53 interaction can occur in living cells we performed co-immunoprecipitation assays. As endogenous TAF6δ is highly labile and expressed at very low levels [5], TAF6δ was expressed by transfection of an expression vector into HCT-116 p53 -/- cells. Exogenous p53 was provided by co-transfection of an expression vector. Immunoprecipitation of TAF6δ resulted in co-immunoprecipitation of p53 (Figure 5C, lane 3), in contrast to the negative control immunoprecipitation of tubulin (Figure 5C, lane 2). In addition, the reciprocal experiment, immunoprecipitation of p53 resulted in recovery of TAF6δ (Figure 5C, lane 4). Together, these data show that the interaction between TAF6δ and p53 can occur in the cellular context.

We next sought to determine whether the interaction between TAF6δ and p53 has functional consequences. We took advantage of the fact that the DUSP1 promoter is activated by TAF6δ (Figure 3B) and is also activated by p53 [33]. A reporter construct expressing firefly luciferase under the control of the DUSP1 promoter was co-transfected with p53 expression vectors, as well as vectors expressing TAF6 variants. TAF6δ transfection resulted in enhanced DUSP1 expression when co-transfected with p53 (Figure 5D). A truncated version of TAF6 that lacks pro-apoptotic activity [4] failed to show significant co-activation (Figure 5D). The protein levels resulting from transfected plasmids were determined by immunoblotting experiments and are shown in Additional File 5. The data exclude the possibility that higher levels of TAF6δ protein (compared to the truncated negative control TAF6) contribute to the activation levels observed. These data show for the first time that TAF6δ can interact functionally with p53 to co-activate DUSP1 gene transcription.

**Figure 6:**
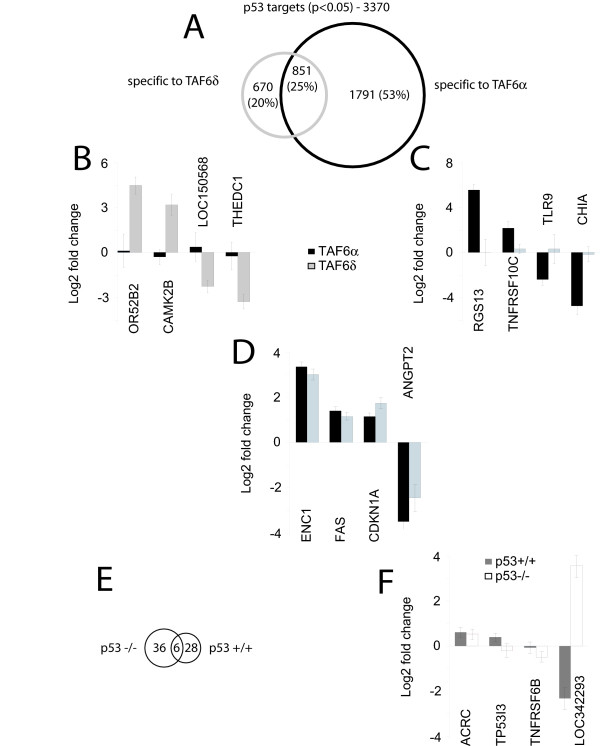
**Evidence for transcriptional crosstalk between endogenous TAF6δ and p53**. (A) Venn diagram illustrating the partitioning of previously characterized p53-regulated genes [5] into TAF6α and TAF6δ dependent classes. (B) Examples of TAF6δ but not TAF6α dependent p53 target genes. Log base 2 fold changes in gene expression are shown graphically. Error bars show the standard deviations over three independent experiments and gene symbols are shown at the bottom. (C) Examples of TAF6α but not TAF6δ dependent p53 target genes (as in panel B). (D) Examples of TAF6α and TAF6δ dependent p53 target genes (as in panel B). (E) Previously characterized TAF6δ target genes [5] partition into p53 dependent and independent genes as illustrated with Venn diagrams. (F) Examples of p53 dependent and independent TAF6δ target genes (as in panel B).

Having established that TAF6δ can interact functionally with p53, we next sought to define the role of this interaction with endogenous TAF6δ and p53. Moreover we sought to define the potential crosstalk of TAF6δ with p53 upon endogenous genes and at the transcriptome-wide level. We therefore revisited microarray data derived from either wild-type HCT-116 or their p53 negative counterpart HCT-116 p53 -/- in which endogenous TAF6δ expression was induced by SSO (http://www.ncbi.nlm.nih.gov/geo/ under accession number GSE10795) to test for transcriptional crosstalk between TAF6δ and p53. We re-filtered the data specifically to determine the influence of TAF6δ versus TAF6α on p53-dependent genes. The effects of TAF6 isoforms are illustrated in Figure 6A, and reveal three classes of genes. Importantly, 20% of p53-regulated genes (e.g. CAMK2B & THEDC1) change only in the presence of TAF6δ (Figure 6B). 53% of the p53-regulated genes change specifically in the presence of TAF6α, for example TNFRSF10C and CHIA (Figure 6C). 25% of p53-regulated genes, such as FAS and ANGPT2, change expression in the presence of both TAF6δ and TAF6α (Figure 6D). The data show that the expression of TAF6δ versus TAF6α can dictate the outcome of p53-mediated transcriptional signals.

We also filtered the data to determine the reciprocal influence of p53 status upon previously identified TAF6δ-dependent mRNAs [5]. 51% of the TAF6δ-regulated mRNAs changed significantly only in the absence of p53 (Figure 6E), for example TNFRSF6B (Figure 6F). 9% of the TAF6δ-regulated mRNAs changed independently of p53 status, including ACRC (Figure 6F). 40% of TAF6δ-regulated mRNAs changed significantly only in cells expressing p53, such as TP53I3 (Figure 6F). In general the influence of p53 on TAF6δ-dependent transcription was relatively subtle in magnitude, with at least one exception where the gene LOC342293 displayed opposing regulation in presence or absence of p53 (Figure 6F). Together, the above data establish reciprocal transcriptional crosstalk between the TAF6δ and p53 proteins.

## Discussion

**Figure 7:**
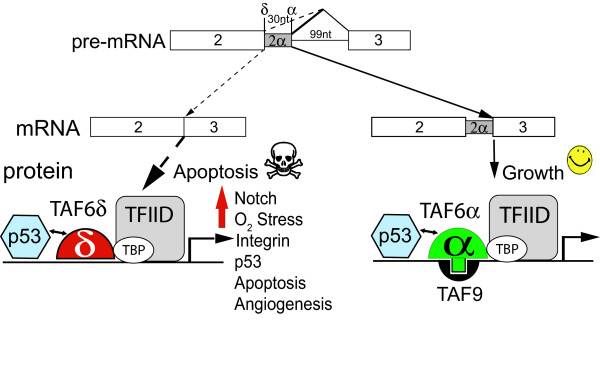
**A model for the TAF6δ pathway**. A hypothetical model coupling changes in gene expression to cell death via the TAF6δ pathway of apoptosis. See Discussion for details.

The TAF6δ pathway has emerged as an apoptotic signaling hub [4,5,21], yet the mechanisms by which TAF6δ promotes apoptosis have remained unknown. Here we provide a transcriptome-wide microarray analysis that defines the impact of endogenous TAF6δ induction on gene expression patterns. The TAF6δ transcriptome footprint showed a predominant role for TAF6δ in the activation of gene expression, with approximately 90% of TAF6δ-regulated mRNAs being induced. Genes annotated as belonging to apoptotic pathways were found to be statistically overrepresented in the TAF6δ-induced genes. The data therefore provide experimental support for a model wherein TAF6δ initiates the apoptotic cascade by inducing pro-apoptotic gene expression. Examples of apoptotic TAF6δ-induced genes identified include NOXA and FDXR (Additional File 3) that both code for proteins that each alone possess pro-apoptotic activity, are p53 target genes, and localize to the mitochondria [35-37]. As TAF6δ induces the expression of hundreds of genes, including established pro-apoptotic genes, we propose a model whereby TAF6δ represents a signaling hub that transduces apoptotic stimuli to redirect the transcriptional machinery to tip the balance from an anti- to a pro-apoptotic program (Figure 7).

In addition to apoptotic genes the unbiased statistical analysis of our microarray data revealed overrepresentation of genes in the Notch, oxidative stress response, integrin, p53, p53 pathway feedback loops 2, and angiogenesis pathways. The TAF6δ pathway is an orphan pathway whose molecular trigger remains unknown. The novel links between these pathways and TAF6δ expression provide testable hypotheses for the potential physiological triggers and functions of TAF6δ. For example, the identification of p53 target genes prompted us to test and demonstrate a physical interaction between TAF6δ and p53 (see below). Within the TAF6δ-activated transcriptome signature, unbiased statistical approach showed significant overrepresented interconnections between these individual signaling pathways (e.g. integrin and angiogenesis). Additional support for functional interconnections amongst TAF6δ-associated pathways comes from interactions documented in the literature. For example, the Notch [38] and integrin [39] pathways both play important roles in angiogenesis. The fact that several of the TAF6δ-induced pathways converge upon the process of angiogenesis implies a physiological coherent impact of TAF6δ on gene expression programs. Interestingly, many of the pathways associated with TAF6δ expression play roles in the tumor progression. For example, angiogenesis is both a key event in tumor progression and a target for anti-cancer therapies [40]. Our study therefore provides the rationale to initiate studies to test the impact of TAF6δ on the process of angiogenesis *in vivo *in the future.

The incorporation of TAF6δ into TAF-containing complexes results in the formation of TFIIDπ that lacks TAF9 (see Introduction). The currently available evidence is consistent with the lack of TAF9 being the only difference between canonical TFIID complexes and TFIIDπ [4], however it is conceivable that the inclusion of TAF6δ could cause as yet unknown changes in TFIIDπ subunit composition. TAF6 interacts with TAF9 and the resulting dimeric complex can bind to downstream promoter elements (DPEs) [41,42]. To date our analysis of TAF6δ-responsive promoters has revealed no statistically significant enrichment of DPEs or any of the known core promoter element within the promoter regions of genes induced by TAF6δ (unpublished data). One mechanistic explanation for the transcriptome impact of TAF6δ could be that the loss of TAF9 or TAF9b from TFIID alone drives transcriptional changes. A prediction of this model is that the transcriptome signatures resulting from depletion of TAF9 and/or TAF9b by small interfering RNAs would be highly similar to that resulting from induction of TAF6δ. The comparative transcriptomic analysis we provide shows both overlapping and unique features of the TAF6δ versus TAF9/TAF9b-dependent transcriptomes. Interestingly, TAF9b-depletion, like TAF6δ induction resulted in transcriptome profile with overrepresentation of genes functioning in angiogenesis pathways. Gene ontology analysis of the genes regulated by all of TAF6δ, TAF9 and TAF9b showed an overrepresentation of a single ontology termed the p53 feedback loops 2 pathway, suggesting overlap in the gene expression programs controlled by these TAFs. A limitation of the current study is that the distinct approaches (siRNA versus SSO RNA) and microarray platforms employed results in the loss of information. Nevertheless, clear differences were observed between the transcriptome profiles of TAF6δ versus TAF9 and TAF9b (Additional File 4). We conclude that exclusion of TAF9 and/or TAF9b from TFIID results in transcriptome changes that share certain targets with, but that do not fully recapitulate the TAF6δ transcriptome signature.

The current transcriptome analysis showed that TAF6δ induces genes in the p53 pathway, a result not revealed by previous transcriptome analysis of TAF6δ in HCT-116 cells [5]. Based on the finding that TAF6δ and p53 can share target genes, we tested and confirmed the direct physical and functional interaction of TAF6δ with p53. The impact of endogenous TAF6δ on p53-dependent gene expression was further demonstrated at the transcriptome-wide level. The reciprocal capacity of endogenous p53 to influence TAF6δ-mediated transcription was also detected, although the magnitude of these effects was globally more modest. Taken together, the data show that there is reciprocal crosstalk between the TAF6δ and p53 pathways. The microarray experiments measure changes in expression resulting from both direct and indirect effects of TAF6δ and p53. Therefore, the crosstalk we have documented includes that resulting from the direct TAF6δ- p53 but also that resulting from indirect transcriptional changes. Given the previous demonstration that TAF6δ can induce apoptosis independent of p53 [5], we conclude that TAF6δ possesses both p53 independent and p53 dependent activities.

## Conclusion

In summary, we report here that the transcriptome landscape orchestrated by TAF6δ includes the induction of apoptotic gene expression. The transcriptome data further uncovered novel links between TAF6δ expression and the Notch, oxidative stress response, integrin, p53, p53 feedback loop 2, and angiogenesis pathways. The TAF6δ-controlled transcriptome landscape was shown not to be equivalent to those resulting from depletion of TAF9 and/or TAF9b. Finally, the data establish a physical and functional interaction between TAF6δ and the p53 tumor suppressor protein.

## Methods

### Cell culture

HeLa cells were grown in DMEM containing 2.5% CS and 2.5% FCS. HCT-116 cells were grown in McCoy's media supplemented with 10% FCS.

### Transfections

2'-O-methyl-oligoribonucleoside phosphorothioate antisense 20-mers were from Sigma-Proligo. "SSO ctrol", "SSO T6-1" [5] and "SSO Bcl-x" [43] have been described. "SSO T6-3" 5'-CUGUGCGAUCUCUUUGAUGC-3' targets the 3' part of the alternative exon 2 of TAF6. SSOs were transfected at a final concentration of 200 nM with lipofectamine 2000 (Invitrogen) as a delivery agent (1.6 μl/ml) according to the manufacturer's recommendations. Plasmids were transfected using 1 μl DMRIE-C (Invitrogen) as a delivery agent in a 24 well plate according to the manufacturer's recommendations. All transfections were performed in OptiMEM medium (Invitrogen).

### Plasmids

Plasmids expressing firefly luciferase under control of the HES1 [29], DUSP1 [33], Bax [44], and ADM [45] promoters have been described. Plasmids expressing TAF6α, TAF6δ and TAF6ΔAB [4] and p53 or its mutated form p53R175H [46] have been described. To construct vectors for bacterial production of His-tagged TAF6α and TAF6δ proteins, full length cDNAs were excised from pXJ42-TAF_II_80α and pXJ42-TAF_II_80δ(ΔA) [4] respectively, using NotI and XhoI sites. The NotI site was filled in by treatment with Klenow enzyme. The generated fragment was inserted into the SalI and Klenow filled HindIII sites of pQE31 vector (Qiagen), generating pQE31-TAF6α and pQE31-TAF6δ plasmids. pGST-p53Arg [47] and pGEX4-T-3 (GE Healthcare) were used for expression of GST-tagged p53 and GST proteins respectively.

### Antibodies

Monoclonal antibodies directed against TAF6δ (37TA-1 & 37TA-2) [4], and TBP (3G3) [48] have been described. The pan-TAF6 monoclonal antibody was purchased from BD Transduction Laboratories. Antibodies against ARNT (sc-17811), JUN (sc-1694), FOS (sc-52), CDKN2B (sc-613) and His probe antibody (sc-803) were purchased from Santa Cruz Biotechnology. Antibodies against HES1 (AB5702), PMAIP1 (Ab13654), alpha-Tubulin (clone B-4-1-2), and p53 (clone PAb1801) were purchased from Millipore, Abcam, Sigma, and Calbiochem respectively.

### RT-PCR

RT-PCR conditions and primers for amplification of both TAF6α and TAF6δ have been described [5].

### Immunocytochemistry

Immunolabelling of TAF6δ in fixed cells was performed as described [5].

### Microarray Analysis of Gene Expression

Transcriptome analysis was performed as we previously detailed [26], using the NeONORM normalization method with k = 0.20 [49]. The published microarray data for TAF9 and TAF9b depletion by siRNA were generated on the Génopole Genomics Platform Strasbourg using custom technology. In order to be able to compare those data directly to data generated from commercial platforms, the unique probe-set identifiers were mapped to non-redundant NCBI and Ensemble gene IDs. Similarly, the AB1700 data generated for this study or from previous studies on an Applied Biosystems Microarray platform were mapped according to the published procedure [50] to the same set of gene IDs. After these mapping procedures >87.9% of unique probe-set or probe IDs could be directly compared which corresponds to > 93.2% comparable genes. For comparative pathway inference analyses the TAF9 and TAF9b data were mapped to, and treated as if AB1700 data to avoid any potential bias stemming from the use of different ontology annotation databases.

Gene Ontology (GO) and KEGG annotations were analyzed using the Panther Protein Classification System http://www.pantherdb.org to identify functional annotations that were significantly enriched in the different gene sets when compared to the whole set of genes present on the ABI microarray. Note that a given gene can be assigned to different pathways; in order to reduce multiple probing biases a gene is weighted by the inverse of the number of pathways it can be assigned to, leading to non-natural numbers for the gene counts. P-values are determined using a binominal distribution and a null hypothesis of a random set of genes with identical size. Pathway interconnectivity analysis was performed for the significantly overrepresented pathways based on genes that are annotated to be part of any combination of two of the selected pathways and that were significantly regulated in the subtraction profile analysis. Those numbers were then compared to the entire set of shared genes, and P-values were calculated as above.

Microarray data for the gene sets analyzed herein are provided as Additional Files 3; 6, 7, 8, 9, 10. The transcriptome-wide microarray data for all of the experiments described here were deposited in the M. ACE database http://mace.ihes.fr under accession numbers:

TAF9/TAF9b: 2833146766

TAF6δ signature: 2937831950; Bcl-x: 2156101006; p53: 2370552334

### Real time PCR

Real time PCR was performed as described [5]. RNA was prepared with using an RNeasy mini Kit (Qiagen). 1 μg of total RNA was reverse transcribed using AMV-RT (Roche). Real-time PCR was performed in a final 25 μl reaction on 10 ng of cDNA with 12.5 μl 2× TaqMan^® ^Universal Master Mix (ABI) and 1.25 μl of the following 20× TaqMan^® ^probes: B2M as the internal control (Hs99999907_m1), ACRC (Hs00369516_m1), ADM (Hs00181605_m1), ATF3 (Hs00231069_m1), DDIT3 (Hs00358796_g1), DUSP1 (Hs00610256_g1), EFNA5 (Hs00157342_m1), HES1 (Hs00172878_m1), HOM-TES-103 (Hs00209961_m1), IFRD1 (Hs00155477_m1), IL6 (Hs00174131_m1), NR4A2 (Hs00428691_m1), PFKFβ4 (Hs00190096_m1), PMAIP1 (Hs00560402_m1), or TRIB3 (Hs00221754_m1).

### Luciferase assays

Cells were washed with PBS and lysed with Passive lysis buffer (Promega). The luciferase activity was measured on a Lumistar luminometer (BMG Labtech), after injection of 2× Luciferin reagent; 270 μM CoenzymeA, 470 μM D-Luciferin, 530 μM ATP (all from Sigma-Aldrich) 40 mM Tris-Phosphate pH 7.8, 2.14 mM MgCl_2_, 5.4 mM MgSO_4_, 0.2 mM EDTA, 33.3 mM DTT.

### Recombinant protein production

Escherichia coli M15 cells were transformed with plasmids expressing His-TAF6α and His-TAF6δ fusion proteins and grown to log-phase before induction of protein expression with 1 mM IPTG (isopropyl-β-d-thiogalactopyranoside) for 18 h at 16.5°C. The cell pellets were resuspended in Ni buffer composed of 1 M NaCl, 30 mM Tris pH8.0 and supplemented with 25% glycerol and 1× Complete protease inhibitor cocktail (PIC) (Roche) and 0.5 mM PMSF (phenylmethyl-sulfonyl fluoride) and sonicated twice for 5 minutes on ice. After clarification, the supernatant was loaded on a His-trap column (GE Healthcare) equilibrated in Ni buffer containing 10 mM imidazole. The column was sequentially washed with Ni buffer containing 60 mM and 100 mM of imidazole and the proteins were finally eluted with 250 mM imidazole. Purified proteins were dialysed against buffer D (20 mM HEPES ph7.9, 100 mM KCl, 20% glycerol, 0.2 mM EDTA).

GST and GST-p53 fusion proteins were produced in Escherichia coli BL-21 cells by IPTG induction (1 mM) of a log-phase culture for 3 h. The cell pellets were resuspended in PBS containing 0.5% Triton X-100 and sonicated twice for 2 minutes. After clarification, the supernatant was incubated with glutathione-sepharose (GE Healthcare) for 1 h 30 at 4°C. The beads were washed three times in PBS and once in buffer D supplemented with 5 mM MgCl_2_, 0.1% NP40 and EDTA up to 1 mM (buffer D+).

### Protein-protein Interactions

GST "pull-down" assays were performed essentially as previously described [17]. GST- and His-tagged proteins were pretreated with 0.5 U DNase (Promega) for 10 minutes at 37°C before the interaction assay. Equal amounts of GST or GST-p53 linked to sepharose beads were then incubated with His-TAF6α, His-TAF6δ or His-TdT for 1 hour at room temperature in buffer D supplemented with 0.5 μg RNase A (USB) (buffer D+). After four washes with buffer D+ containing 100 mM or 600 mM KCl, bound proteins were analyzed by SDS-PAGE and immunoblotting.

### Immunoprecipitation

Cells were lysed in RIPA buffer (50 mM Tris pH8, 1% NP40, 0.25% Na deoxycholate, 150 mM KCl, 1 mM EDTA) supplemented with 1× PIC and 0.5 mM PMSF. The lysate was diluted 1/10 with IP100 buffer (25 mM Tris pH8, 5 mM MgCl2, 10% glycerol, 100 mM KCl, 0.1% NP40, 0.3 mM DTT, PIC, PMSF) and precleared with proteinG-sepharose beads for 2 hours at 4°C. The precleared lysate was then incubated overnight at 4°C with anti-p53, anti-TAF6δ or anti-tubulin antibodies immobilized on protein G-sepharose beads. After extensive washes with IP100 buffer, complexes were analyzed by SDS-PAGE and immunoblotting.

## Authors' contributions

EW performed or directed all of the experiments with the exception of microarray analysis. MK performed the reporter gene experiments. BT and JVE performed and analysed the microarray data. MF and LT generated and provided microarray data on TAF9/TAF9b. BB and AB conceived and designed the experiments and wrote the manuscript. All the authors read and approved the final manuscript.

## Supplementary Material

Additional file 1 **Splice-switching oligonucleotide (SSO) targeting of TAF6**. (A) The region of the TAF6 pre-mRNA that includes two alternative 5' splice sites (SSs) that produce either the constitutive α splice variant or the alternative δ splice variant is schematically depicted. Selection of an intron-proximal α 5' splice site (SS) results in production of the α isoform of TAF6 (at right) whereas the selection of the proximal δ 5' SS results in the production of the δ isoform (at left). The SSO oligonucleotides (SSO T6-1 & SSO T6-3) base pair with the alternative exon to force splicing from the distal 5' SS and enforce expression of the endogenous TAF6δ isoform (at left). The protein produced by the major splice variant, TAF6α, can interact with the TFIID subunit, TAF9 via its histone fold domain. In contrast, TAF6δ lacks 10 amino acids of helix 2 of its histone fold motif and therefore cannot interact with TAF9. (B) SSO T6-3 induces endogenous TAF6δ mRNA expression. HeLa cells were transfected with antisense oligonucleotides: SSO Ctrol, SSO T6-1 or SSO T6-3. 24 hours post-transfection total RNA was isolated and subjected to RT-PCR with primers that amplify both the TAF6α and the alternative TAF6δ mRNAs. (C) Quantification of TAF6δ expression. Black bars show the percentage of TAF6δ over total TAF6 mRNA as amplified by RT-PCR as in B and separated by microfluidity and analyzed using a 2100 Agilent Bioanalyzer. Light grey bars show the percentage of TAF6δ expressing cells after SSO transfection as in B except that cells were fixed and stained with an anti-TAF6δ antibody for immunocytochemistry (ICC) and a minimum of 500 cells were scored for their staining with an anti-TAF6δ antibody. Click here for file

Additional file 2 **The transcriptome impact of TAF6δ-inducing splice switching oligonucleotides (SSO) is highly distinct from the impact of Bcl-x SSO**. TAF6δ-inducing SSO microarray data have been compared with previously documented Bcl-x SSO data [26]. Venn diagrams show the number of distinct and overlapping genes in the up-regulated (top) or down-regulated (bottom) gene subsets resulting from treatment with TAF6δ versus Bcl-x SSO. Click here for file

Additional file 3 **TAF6δ Signature.csv**. This comma separated value data file contains a tabular listing of the TAF6δ target genes identified in this study and their annotation. Click here for file

Additional file 4 **Differential regulation of gene expression by TAF6δ versus TAF9/TAF9b**. Log base 2 fold changes in gene expression are represented by black (TAF6δ-regulated), white bars (TAF9-regulated), or grey (TAF9b-regulated) bars. Error bars show the standard deviations over three independent experiments and gene symbols are shown at the bottom. Click here for file

Additional file 5 **Proteins levels resulting from transfection of TAF6 and p53**. HeLa cells were transfected with plasmids expressing TAF6, p53 or p53 bearing the R175H mutation, and DUSP1-luciferase reporter constructs. (A) Total protein extracts were prepared 28 hours post-transfection and fractionated by SDS-PAGE followed by immunoblot analysis of exogenous protein levels with antibodies indicated at the left. A representative immunoblot is shown. (B) Protein levels from three independent transfections were quantitated by phosphoimager analysis and normalized to Actin levels. Error bars show the standard deviations. Click here for file

Additional file 6 **TAF9 Signature.csv**. This comma separated value data file contains a tabular listing of the TAF9 target genes identified in this study and their annotation. Click here for file

Additional file 7 **TAF9b Signature.csv**. This comma separated value data file contains a tabular listing of the TAF9b target genes identified in this study and their annotation. Click here for file

Additional file 8 **TAF6δ TAF9 common genes.csv**. This comma separated value data file contains a tabular listing of the common TAF6δ and TAF9 target genes identified in this study and their annotation. Click here for file

Additional file 9 **TAF6δ TAF9b common genes.csv**. This comma separated value data file contains a tabular listing of the common TAF6δ and TAF9b target genes identified in this study and their annotation. Click here for file

Additional file 10 **TAF6δ TAF9 TAF9b common genes.csv**. This comma separated value data file contains a tabular listing of the common TAF6δ and TAF9 and TAF9b target genes identified in this study and their annotation. Click here for file

## References

[B1] Hengartner MO The biochemistry of apoptosis Nature 2000 407 6805 770 776 10.1038/35037710 11048727

[B2] Thompson CB Apoptosis in the pathogenesis and treatment of disease Science 1995 267 5203 1456 1462 10.1126/science.7878464 7878464

[B3] Reed JC Apoptosis-based therapies Nat Rev Drug Discov 2002 1 2 111 121 10.1038/nrd726 12120092

[B4] Bell B Scheer E Tora L Identification of hTAF(II)80 delta links apoptotic signaling pathways to transcription factor TFIID function Mol Cell 2001 8 3 591 600 10.1016/S1097-2765(01)00325-2 11583621

[B5] Wilhelm E Pellay FX Benecke A Bell B TAF6delta controls apoptosis and gene expression in the absence of p53 PLoS ONE 2008 3 7 e2721 10.1371/journal.pone.0002721 18628956 PMC2444026

[B6] Bell B Tora L Regulation of gene expression by multiple forms of TFIID and other novel TAFII-containing complexes Exp Cell Res 1999 246 1 11 19 10.1006/excr.1998.4294 9882510

[B7] Green MR TBP-associated factors (TAFIIs): multiple, selective transcriptional mediators in common complexes Trends Biochem Sci 2000 25 2 59 63 10.1016/S0968-0004(99)01527-3 10664584

[B8] Muller F Demeny MA Tora L New problems in RNA polymerase II transcription initiation: matching the diversity of core promoters with a variety of promoter recognition factors J Biol Chem 2007 282 20 14685 14689 10.1074/jbc.R700012200 17395580

[B9] Weinzierl RO Ruppert S Dynlacht BD Tanese N Tjian R Cloning and expression of Drosophila TAFII60 and human TAFII70 reveal conserved interactions with other subunits of TFIID Embo J 1993 12 13 5303 5309 8262073 10.1002/j.1460-2075.1993.tb06226.x PMC413796

[B10] Wright KJ Marr MT Tjian R TAF4 nucleates a core subcomplex of TFIID and mediates activated transcription from a TATA-less promoter Proc Natl Acad Sci USA 2006 103 33 12347 12352 10.1073/pnas.0605499103 16895980 PMC1567882

[B11] Selleck W Howley R Fang Q Podolny V Fried MG Buratowski S Tan S A histone fold TAF octamer within the yeast TFIID transcriptional coactivator Nat Struct Biol 2001 8 8 695 700 10.1038/90408 11473260

[B12] Michel B Komarnitsky P Buratowski S Histone-like TAFs are essential for transcription in vivo Mol Cell 1998 2 5 663 673 10.1016/S1097-2765(00)80164-1 9844638

[B13] Hisatake K Ohta T Takada R Guermah M Horikoshi M Nakatani Y Roeder RG Evolutionary conservation of human TATA-binding-polypeptide-associated factors TAFII31 and TAFII80 and interactions of TAFII80 with other TAFs and with general transcription factors Proc Natl Acad Sci USA 1995 92 18 8195 8199 10.1073/pnas.92.18.8195 7667268 PMC41123

[B14] Xie X Kokubo T Cohen SL Mirza UA Hoffmann A Chait BT Roeder RG Nakatani Y Burley SK Structural similarity between TAFs and the heterotetrameric core of the histone octamer Nature 1996 380 6572 316 322 10.1038/380316a0 8598927

[B15] Chen Z Manley JL In vivo functional analysis of the histone 3-like TAF9 and a TAF9-related factor, TAF9L J Biol Chem 2003 278 37 35172 83 10.1074/jbc.M304241200 12837753

[B16] Frontini M Soutoglou E Argentini M Bole-Feysot C Jost B Scheer E Tora L TAF9b (formerly TAF9L) is a bona fide TAF that has unique and overlapping roles with TAF9 Mol Cell Biol 2005 25 11 4638 4649 10.1128/MCB.25.11.4638-4649.2005 15899866 PMC1140618

[B17] Thut CJ Chen JL Klemm R Tjian R p53 transcriptional activation mediated by coactivators TAFII40 and TAFII60 Science 1995 267 5194 100 104 10.1126/science.7809597 7809597

[B18] Liu WL Coleman RA Ma E Grob P Yang JL Zhang Y Dailey G Nogales E Tjian R Structures of three distinct activator-TFIID complexes Genes Dev 2009 23 13 1510 1521 10.1101/gad.1790709 19571180 PMC2704470

[B19] Farmer G Colgan J Nakatani Y Manley JL Prives C Functional interaction between p53, the TATA-binding protein (TBP), andTBP-associated factors in vivo Mol Cell Biol 1996 16 8 4295 4304 8754830 10.1128/mcb.16.8.4295 PMC231428

[B20] Jimenez GS Nister M Stommel JM Beeche M Barcarse EA Zhang XQ O'Gorman S Wahl GM A transactivation-deficient mouse model provides insights into Trp53 regulation and function Nat Genet 2000 26 1 37 43 10.1038/79152 10973245

[B21] Gill G Death signals changes in TFIID Mol Cell 2001 8 3 482 484 10.1016/S1097-2765(01)00338-0 11583609

[B22] Aviel-Ronen S Coe BP Lau SK da Cunha Santos G Zhu CQ Strumpf D Jurisica I Lam WL Tsao MS Genomic markers for malignant progression in pulmonary adenocarcinoma with bronchioloalveolar features Proc Natl Acad Sci USA 2008 105 29 10155 10160 10.1073/pnas.0709618105 18632575 PMC2465804

[B23] Campbell JM Lockwood WW Buys TP Chari R Coe BP Lam S Lam WL Integrative genomic and gene expression analysis of chromosome 7 identified novel oncogene loci in non-small cell lung cancer Genome 2008 51 12 1032 1039 10.1139/G08-086 19088816

[B24] Dressman HK Hans C Bild A Olson JA Rosen E Marcom PK Liotcheva VB Jones EL Vujaskovic Z Marks J Dewhirst MW West M Nevins JR Blackwell Kl Gene expression profiles of multiple breast cancer phenotypes and response to neoadjuvant chemotherapy Clin Cancer Res 2006 12 3 Pt 1 819 826 10.1158/1078-0432.CCR-05-1447 16467094

[B25] Wang W Nahta R Huper G Marks JR TAFII70 isoform-specific growth suppression correlates with its ability to complex with the GADD45a protein Mol Cancer Res 2004 2 8 442 452 15328371

[B26] Wilhelm E Pellay FX Benecke A Bell B Determining the impact of alternative splicing events on transcriptome dynamics BMC Res Notes 2008 1 94 10.1186/1756-0500-1-94 18950505 PMC2584107

[B27] Batagelj V Mrvar A Mutzel P, Jünger M, Leipert S Pajek - Analysis and Visualization of Large Networks Graph Drawing: 9th International Symposium, Gd 2001, Vienna, Austria, September 23-26, 2001, Revised Papers 2002 Berlin, Heidelberg: Springer 477 478 [Lecture Notes in Computer Science, vol. 2265]

[B28] Oda E Ohki R Murasawa H Nemoto J Shibue T Yamashita T Tokino T Taniguchi T Tanaka N Noxa, a BH3-only member of the Bcl-2 family and candidate mediator of p53-induced apoptosis Science 2000 288 5468 1053 1058 10.1126/science.288.5468.1053 10807576

[B29] Jarriault S Brou C Logeat F Schroeter EH Kopan R Israel A Signalling downstream of activated mammalian Notch Nature 1995 377 6547 355 358 10.1038/377355a0 7566092

[B30] Nikitenko LL Fox SB Kehoe S Rees MC Bicknell R Adrenomedullin and tumour angiogenesis Br J Cancer 2006 94 1 1 7 10.1038/sj.bjc.6602832 16251875 PMC2361077

[B31] Zhou JY Liu Y Wu GS The role of mitogen-activated protein kinase phosphatase-1 in oxidative damage-induced cell death Cancer Res 2006 66 9 4888 4894 10.1158/0008-5472.CAN-05-4229 16651445

[B32] Liu YX Wang J Guo J Wu J Lieberman HB Yin Y DUSP1 is controlled by p53 during the cellular response to oxidative stress Mol Cancer Res 2008 6 4 624 633 10.1158/1541-7786.MCR-07-2019 18403641

[B33] Li M Zhou JY Ge Y Matherly LH Wu GS The phosphatase MKP1 is a transcriptional target of p53 involved in cell cycle regulation J Biol Chem 2003 278 42 41059 41068 10.1074/jbc.M307149200 12890671

[B34] Miyashita T Reed JC Tumor suppressor p53 is a direct transcriptional activator of the human bax gene Cell 1995 80 2 293 299 10.1016/0092-8674(95)90412-3 7834749

[B35] Hwang PM Bunz F Yu J Rago C Chan TA Murphy MP Kelso GF Smith RA Kinzler KW Vogelstein B Ferredoxin reductase affects p53-dependent, 5-fluorouracil-induced apoptosis in colorectal cancer cells Nat Med 2001 7 10 1111 1117 10.1038/nm1001-1111 11590433 PMC4086305

[B36] Liu G Chen X The ferredoxin reductase gene is regulated by the p53 family and sensitizes cells to oxidative stress-induced apoptosis Oncogene 2002 21 47 7195 7204 10.1038/sj.onc.1205862 12370809

[B37] Yu J Zhang L Hwang PM Kinzler KW Vogelstein B PUMA induces the rapid apoptosis of colorectal cancer cells Mol Cell 2001 7 3 673 682 10.1016/S1097-2765(01)00213-1 11463391

[B38] Phng LK Gerhardt H Angiogenesis: a team effort coordinated by notch Dev Cell 2009 16 2 196 208 10.1016/j.devcel.2009.01.015 19217422

[B39] Serini G Valdembri D Bussolino F Integrins and angiogenesis: a sticky business Exp Cell Res 2006 312 5 651 658 10.1016/j.yexcr.2005.10.020 16325811

[B40] Kerbel RS Tumor angiogenesis N Engl J Med 2008 358 19 2039 2049 10.1056/NEJMra0706596 18463380 PMC4542009

[B41] Burke TW Kadonaga JT The downstream core promoter element, DPE, is conserved from Drosophila to humans and is recognized by TAFII60 of Drosophila Genes Dev 1997 11 22 3020 3031 10.1101/gad.11.22.3020 9367984 PMC316699

[B42] Shao H Revach M Moshonov S Tzuman Y Gazit K Albeck S Unger T Dikstein R Core promoter binding by histone-like TAF complexes Mol Cell Biol 2005 25 1 206 219 10.1128/MCB.25.1.206-219.2005 15601843 PMC538770

[B43] Mercatante DR Bortner CD Cidlowski JA Kole R Modification of alternative splicing of Bcl-x pre-mRNA in prostate and breast cancer cells. analysis of apoptosis and cell death J Biol Chem 2001 276 19 16411 16417 10.1074/jbc.M009256200 11278482

[B44] Gaiddon C Moorthy NC Prives C Ref-1 regulates the transactivation and pro-apoptotic functions of p53 in vivo Embo J 1999 18 20 5609 5621 10.1093/emboj/18.20.5609 10523305 PMC1171629

[B45] Ishimitsu T Miyata A Matsuoka H Kangawa K Transcriptional regulation of human adrenomedullin gene in vascular endothelial cells Biochem Biophys Res Commun 1998 243 2 463 470 10.1006/bbrc.1998.8110 9480831

[B46] Hinds PW Finlay CA Quartin RS Baker SJ Fearon ER Vogelstein B Levine AJ Mutant p53 DNA clones from human colon carcinomas cooperate with ras in transforming primary rat cells: a comparison of the "hot spot" mutant phenotypes Cell Growth Differ 1990 1 12 571 580 2288874

[B47] Thomas M Kalita A Labrecque S Pim D Banks L Matlashewski G Two polymorphic variants of wild-type p53 differ biochemically and biologically Mol Cell Biol 1999 19 2 1092 1100 9891044 10.1128/mcb.19.2.1092 PMC116039

[B48] Brou C Wu J Ali S Scheer E Lang C Davidson I Chambon P Tora L Different TBP-associated factors are required for mediating the stimulation of transcription in vitro by the acidic transactivator GAL-VP16 and the two nonacidic activation functions of the estrogen receptor Nucleic Acids Res 1993 21 1 5 12 10.1093/nar/21.1.5 8441620 PMC309058

[B49] Noth S Brysbaert G Benecke A Normalization using weighted negative second order exponential error functions (NeONORM) provides robustness against asymmetries in comparative transcriptome profiles and avoids false calls Genomics Proteomics Bioinformatics 2006 4 2 90 109 10.1016/S1672-0229(06)60021-1 16970549 PMC5054038

[B50] Noth S Benecke A Avoiding inconsistencies over time and tracking difficulties in Applied Biosystems AB1700/Panther probe-to-gene annotations BMC Bioinformatics 2005 6 307 10.1186/1471-2105-6-307 16372901 PMC1361791

